# A Handful of Details to Ensure the Experimental Reproducibility on the FORCED Running Wheel in Rodents: A Systematic Review

**DOI:** 10.3389/fendo.2021.638261

**Published:** 2021-05-10

**Authors:** Daniel Garrigos, Marta Martínez-Morga, Angel Toval, Yevheniy Kutsenko, Alberto Barreda, Bruno Ribeiro Do Couto, Fernando Navarro-Mateu, José Luis Ferran

**Affiliations:** ^1^ Department of Human Anatomy and Psychobiology, Faculty of Medicine, University of Murcia, Murcia, Spain; ^2^ Institute of Biomedical Research of Murcia—IMIB, Virgen de la Arrixaca University Hospital, University of Murcia, Murcia, Spain; ^3^ Faculty of Psychology, University of Murcia, Murcia, Spain; ^4^ Unidad de Docencia, Investigación y Formación en Salud Mental (UDIF-SM), Servicio Murciano de Salud, Murcia, Spain; ^5^ CIBER de Epidemiología y Salud Pública (CIBERESP), Madrid, Spain; ^6^ Departamento de Psicología Básica y Metodología, Universidad de Murcia, Murcia, Spain

**Keywords:** animal research, forced exercise, forced wheel, research guidelines, rodent exercise

## Abstract

A well-documented method and experimental design are essential to ensure the reproducibility and reliability in animal research. Experimental studies using exercise programs in animal models have experienced an exponential increase in the last decades. Complete reporting of forced wheel and treadmill exercise protocols would help to ensure the reproducibility of training programs. However, forced exercise programs are characterized by a poorly detailed methodology. Also, current guidelines do not cover the minimum data that must be included in published works to reproduce training programs. For this reason, we have carried out a systematic review to determine the reproducibility of training programs and experimental designs of published research in rodents using a forced wheel system. Having determined that most of the studies were not detailed enough to be reproducible, we have suggested guidelines for animal research using FORCED exercise wheels, which could also be applicable to any form of forced exercise.

## Introduction

Improving the way of reporting animal research is an uphill task that involves the joint effort of authors, editors, reviewers, funding agencies and the painstaking work of ethics committees (1–3). However, the lack of transparency in reporting research methods remains a concern (1, 4–6). Detailed descriptions of methods and experimental designs might be decisive in minimizing unnecessary experimental duplication and ensuring the reproducibility of research results (3, 7, 8). In the long term, inappropriate descriptions of methods might contribute to diminish the credibility and sustainability of the scientific system and damage the reputation of the researchers involved in these studies (3, 9, 10). Increased evidence indicates that there is an appreciable room for improving the ‘reproducibility crisis’ in science (10–12). In this sense, the Animal Research Reporting of In Vivo Experiments (ARRIVE) guidelines were developed for reporting animal research rigorously, providing checklists with the essential information that must be included in research with animal experimentation (3, 6). However, to reproduce an exercise program in rodents, several additional specifications that are not included in any of the current guidelines are required.

Animal research with voluntary or forced exercise reporting the positive impact of physical activity in health is skyrocketing (13–17). Forced treadmill and wheel exercise, but not voluntary, are the preferred modalities to develop the same training in all animals. Specifically, forced wheel is an emergent modality that should ensure the exercise load reproducibility and avoid non-specific stress-related responses (18, 19), but methods provided in most of these works do not seem to be properly detailed. In this sense, current guidelines do not guarantee the reproducibility of the training programs in forced wheel or any other forced modality (6). Therefore, to allow the repeatability of the same experimental design, giving longevity to the research works using forced wheel, reporting accurate information about the ethic committee, housing, husbandry, animals and particularly exercise programs becomes essential (20–27). Furthermore, physical capacity or exercise adaptations promoted by training programs might be affected by housing or husbandry variations in the environmental conditions, food composition, the number of animals per cage or the light/dark cycle period (21, 28, 29). Moreover, the biological and behavioral research output might change if the animals exposed to the same exercise load present differences in terms of age, sex, weight or handling procedures (20, 23, 27, 30).

Particularly in exercise, variables such as habituation, duration of the session, speed, frequency and duration of the exercise program are determinant to guarantee the reproducibility of the forced wheel training (18, 19, 31). Finally, reporting the time elapsed between the last session of exercise and the test or biological analysis should avoid circadian and metabolic misinterpretations (32–34). A large number of studies have proposed the health benefits of exercise (17, 35–37). However, these conclusions could be called into question if the variables affecting the response to exercise have not been adequately controlled. Rodent research in the exercise field, would be benefited if most of these demands were included in the published works.

Our systematic review aimed to evaluate the reproducibility of the training programs and experimental designs of studies using a forced wheel system. Data ensuring the reproducibility of a training program and those that might potentially affect the physical response were selected and arranged in four sections: ethic committee, housing, animals, and exercise. Our results have led us to suggest the FORCED exercise wheel guidelines to improve the reliability of scientific works based on forced training programs. Our FORCED guidelines complement current guidelines by focusing in forced exercise (3, 6). As a novelty, the number of items reached by research works dealing with forced exercise was analyzed.

## Methods

### Literature Search Strategy

**Figure 1:**
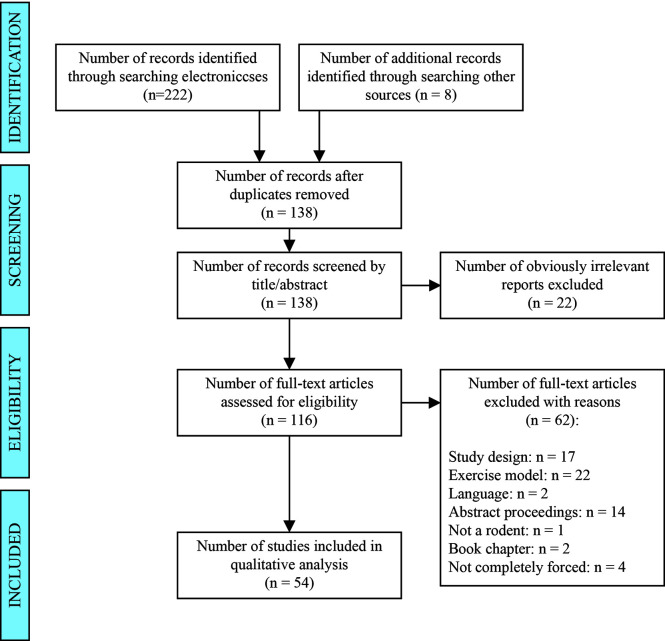
PRISMA flow diagram of study selection.

This systematic review was reported following the Preferred Reporting Items for Systematic Reviews and Meta-analysis (PRISMA, see **Supplementary File 1**) (38). The search was based in two groups of keywords: (i) forced wheel (“forced running wheel*” OR “forced wheel” OR “motorized wheel*”) and (ii) rodents (rodent* OR rat OR rats OR mice OR mouse OR mus OR murine). No additional filters were applied since one of the main objectives of the review was to scope all the available literature. The search was conducted using four electronic bibliographic databases: PubMed, Web of Science, Scopus and Science Direct. The last search was performed on March 23^rd^, 2020. Detailed examples of the search strategy can be found in supplementary materials (see **Supplementary File 4**). Reference lists of all the included papers and relevant reviews were scanned to identify any additional studies manually (Identification, **Figure 1**).

### Study Eligibility Criteria

First screening of obviously irrelevant studies was conducted by two reviewers independently. Resultant full-text studies were then independently assessed using the inclusion and exclusion criteria, which were defined before the screening phase. Any disagreements between the reviewers were resolved by consensus after discussion with a third author. Articles were included in this review if they (1): were published in English (2), intervened on rodents (3), performed forced exercise in a motorized wheel and (4) the study design was a Randomized Controlled Trial or quasi-experimental. Articles were excluded if (1): Only voluntary exercise was performed (2), the forced motorized wheel system implies some additional stimulus to maintain the race. (Screening and eligibility, **Figure 1**).

For the purpose of this review, it was considered forced exercise when the three main parameters of the load (i.e. volume, intensity and density) were forced. In addition, interventions in which the rodent was manually forced to run on a wheel were not considered forced exercise, since the aim of the review focuses on forced exercise from the movement in a motorized wheel. These studies were discarded in the full-text review phase (**Figure 1**).

### Data Extraction and Management

A data extraction form was elaborated before the fieldwork started (See **Supplementary File 5** for detailed extraction protocol). The data extracted from the included studies were categorized in four sections: (i) Animal ethic committee (ii) Housing (iii) Animals and (iv) Exercise. Data extraction was conducted by two authors independently and disagreements were resolved by consensus with a third author.

### Risk of Bias Assessment

Bias was assessed using SYRCLE’s risk of bias tool (39). According to the SYRCLE’s guidelines, the symbol ‘+’ was used when the criteria was reported by the authors. The symbol ‘-’ was used when the item was not reported and ‘?’ was used to indicate that the criteria was unclear. These procedures were conducted by two reviewers independently and any discrepancies were resolved by consensus with a third reviewer. The risk of bias establishment was done in an informative way. The data obtained were not used to establish any measure of treatment effect as it was not the aim of this review.

### Analysis and Statistics

After data extraction, the number of articles reporting or not each item was calculated as a percentage. To determine the distribution of the studies according to the items described, a frequency histogram was used. The Pearson’s correlation coefficient (r) was used to examine the relationship between the quality of the study and i) the year of publication and ii) current impact factor of the journal in which it was published. The r value was rated as trivial (< 0.10), small (0.10–0.29), moderate (0.30–0.49), large (0.50–0.69), very large (0.70–0.89), or nearly perfect (0.90–0.99) (40). P-value was considered statistically significant at p<0.05. Due to the heterogeneity of topics among the studies that met our inclusion criteria, it was not considered appropriate to perform a meta-analysis of the trials.

## Results and Discussion

### Study Eligibility

The flow chart of selected studies is shown in **Figure 1**. A total of 222 studies were found trough database search and 8 studies from manual search. After eliminating duplicates and screening by title and abstracts, 116 studies were assessed for eligibility. Finally, 54 studies met our inclusion criteria.

### Characteristics of the Studies

**Supplementary File 2** describes a summary of the rodent model and the exercise program used in each study included in this review. Rats were the rodents used in 75.9% (41/54) of the studies (18, 41–80), and mice in 24.1% (13/54) (81–93). In rats, the most frequent strain was Sprague-Dawley (22/54) (18, 43, 46–49, 51–55, 61, 64–67, 70, 71, 76, 77, 79, 80), followed by Wistar (10/54) (44, 45, 57–60, 63, 68, 69, 73). In mice, the most frequent strain was C57BL/6J (10/54) (81, 82, 84–89, 92, 93) and BALB/c (2/54) (83, 90) in second place. Most of the studies used healthy animals (22/54) (18, 41, 44, 48, 49, 53, 56, 57, 59, 60, 68, 69, 72–75, 78, 82, 84, 85, 92), followed by stroke models (5/54) (45, 64, 79, 88, 89). Relative to exercise frequency, 5 days per week was the most common weekly frequency and 1 session per day was the most common daily frequency.

### Risk of Bias Assessment

**Figure 2:**
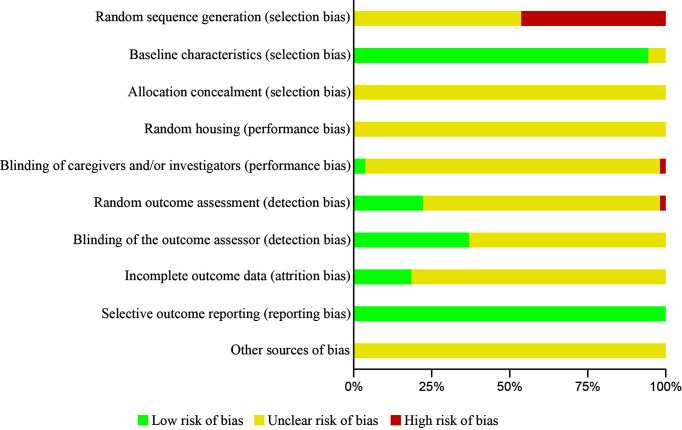
Risk of bias assessment using SYRCLE’s tool. Author’s judgment presented as percentage on each item. (-) High risk of bias, eminent risk of bias for this item; (?) Unclear risk of bias, carefully check the article for this item interpretation; (+) Low risk of bias, free of risk of bias in this item.

A summary of the risk of bias of the studies can be found in **Figure 2**. Since the review covered a wide variety of topics, a conservative and general analysis was decided when assessing the baseline characteristics (item 2, **Figure 2**). Low risk of bias was established if (i) the genetic modification of the animal, (ii) the sex of the sample, and (iii) the age or weight of the sample were described. In case there was no genetic modification: sex, age and weight should be described. When some of these items were described, but not all, it was considered as an unclear risk of bias. High risk was considered when none of the items, or only the sex was mentioned. In none of the studies was it possible to find a high or low risk in the allocation concealment or random housing (items 3 and 4, **Figure 2**). It was not possible to find other potential risks besides those included in the SYRCLE’s tool.

### Animal Ethics Committees

**Figure 3:**
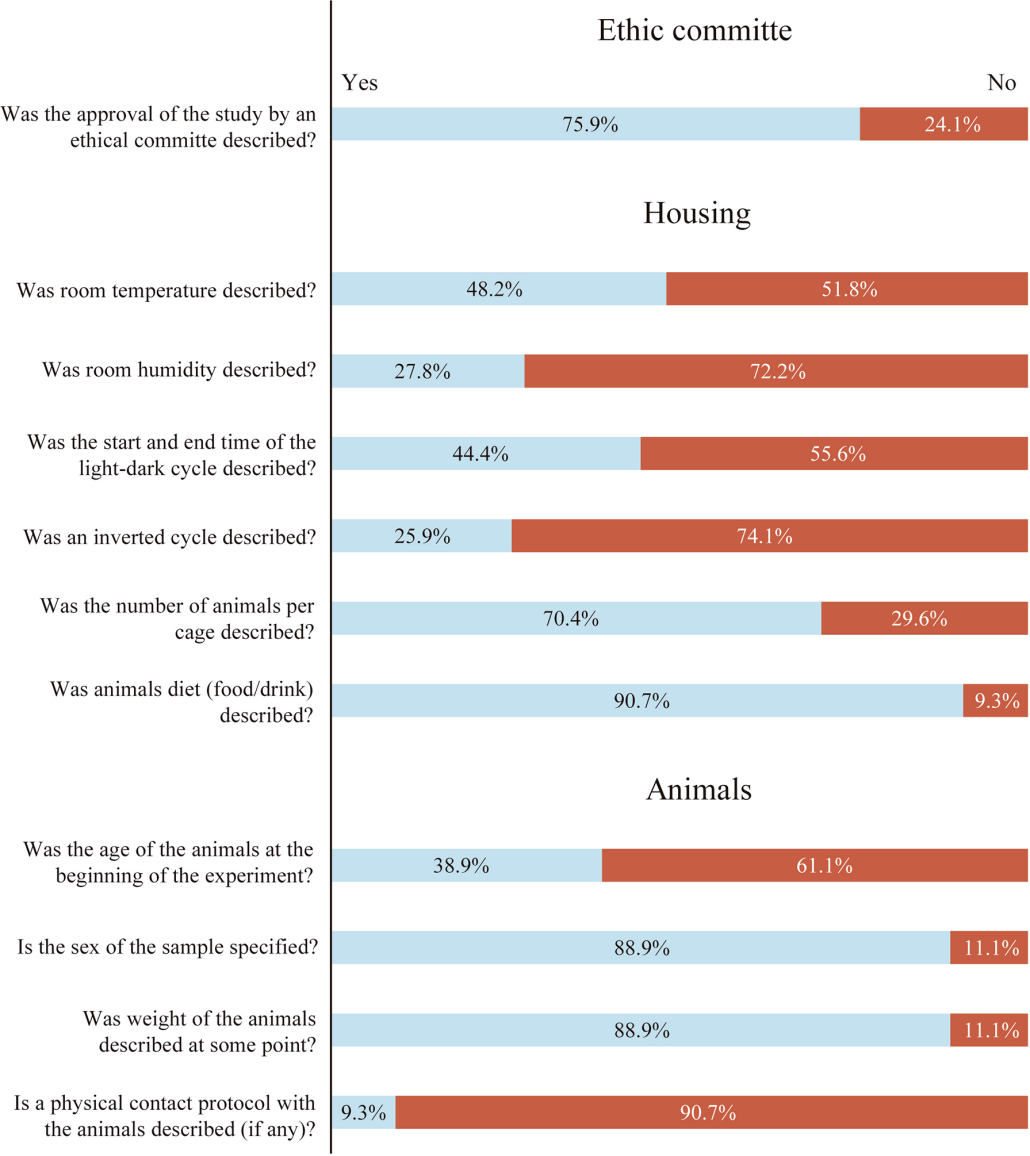
Percentage of studies that reported each item in relation to Ethics Committee, Housing and Animals sections. Light blue box: Item reported percentage. Red box: Item not reported percentage.

Almost 76% (41/54) of the studies analyzed described that the experimental procedures were evaluated and approved by an ethics committee (**Figure 3**). The first animal ethics committees were established during the 70s to review the acceptability of animal research during the experimental procedures, deriving in the 3Rs principles (Replacement, Reduction and Refinement) (2, 94). However, an international consensus of ethical review began 20 years ago, being established as a mandatory requirement for the last 10 years (94, 95). Currently, these committees decide about the ethical acceptability of a research proposal and the researchers’ behavior during the procedures. Singularly, 46.1% (6/13) of the studies that did not describe any ethical committee approval were developed before the international consensus; but 53.8% (7/13) of these works were performed in a period of high consensus.

### Housing

Changes in environmental temperature or humidity, light/dark cycle, number of animals per cage or food and drink availability in the rodents might modify the output results.

#### Room Temperature

In our analysis, the room temperature was described only in 48.2% (26/54) of the papers (**Figure 3**). The thermoneutral zone in mice is usually registered at 30°C, within a range of 1-3°C (96). Thus, mice housed at conventional environmental temperatures (~22°C) are below thermoneutrality; and the maintenance of the core body temperature at these conditions requires about half of the total energy expenditure (97). Only a few degrees of variation in the environmental temperature can change the thermogenesis response, as well as metabolic variations related to lipogenesis, adipogenesis or insulin sensitivity (26). Furthermore, temperature fluctuations can affect the cardiovascular response of the animal to carry out the programmed exercise, thus altering the response and the experimental results (21, 98, 99). Given all this, environmental temperature details result mandatory in all published works involving rodent models (100–102).

#### Environmental Relative Humidity

Unexpectedly, only 27.8% (15/54) of the studies included in our analysis described the room humidity conditions (**Figure 3**). Fluctuations of relative humidity may contribute to the development of dermal diseases or facilitate the transmission of certain viruses (103, 104). Also, developing an exercise program in a hot and high humidity environment can cause an inflammatory response as well as tissue damage (e.g. liver injury) affecting the internal load carried by the animal during the exercise period (25).

#### Light/Dark Cycle

In this review, only 44.5% (24/54) described the starting time of the light/dark cycles and a few studies gave precise data about the beginning time of the experiment (See exercise section). Interestingly, it was observed that 74% (18/24) of the reports describing the light-dark cycle developed the experimental phase during the passive period (light phase), but in most of them no justification was found for the chosen period (**Figure 3**). Most of the rodent models used in experimental research are active during the dark phase (nocturnal) and few of them are active during the light phase (diurnal) (105). Light is the major synchronizer of the circadian rhythms by the action of suprachiasmatic nucleus and peripheral clocks, deriving in different metabolic responses throughout the day (106–108). Also, the disruption of these circadian rhythms can lead to altered cardiovascular, metabolic and neurological responses (32–34, 93). Given this, for an adequate data comparison and reproducibility of the experiment, it is crucial to know the parameters of the light/dark cycle (active or passive) under which the interventions were carried out (28). However, for most of the papers analyzed, it was difficult -or even not possible- to know the period in which the experiment was developed; a situation that can be surpassed by including the start time and duration of the light/dark cycle and the start-end time of the experiments.

#### Animals per Cage

The number of animals per cage was indicated in 70.4% (38/54) of the studies analyzed (**Figure 3**). Rodents are social animals, and it is well known that their isolation can modify the experimental results. As a chronic stressor, individual housing can have strong effects on behavior, leading to stereotyped behavior or provoking depression- and anxiety-like symptoms (109, 110). This chronic stress involves structural and molecular alterations in several areas of the brain, particularly the prefrontal cortex and limbic brain structures, deriving in altered psychotic and emotional behavior (111–113). Executing a physical exercise program may mitigate, but not replace the beneficial effects of social interaction (114). It is crucial to report the number of animals per cage, and the sex of the animal (See below), since it can condition other social aspects such as the establishment of hierarchies or dominance behaviors that may affect the results.

#### Food/Drink

The 90.7% (49/54) of the studies analyzed described the type of food/drink and its availability (**Figure 3**). Interestingly, 36 of them consisted of a standard ad libitum food/drink consumption, while 13 studies introduced some type of modification of these parameters. Experimental designs with differences in food and water macronutrients composition (fat, protein and carbohydrate percentages) and in its access by the rodent, may strongly affect the experimental outputs (29, 115, 116). Some macronutrient proportions may lead to white adipose tissue inflammation, insulin resistance or obesity (117, 118). On the other hand, changes in intake patterns will derive in a variation of circadian hormones (e.g. insulin, glucagon or corticosterone) (119, 120); or may modify the expression of intestinal enzymes such as maltose and sucrose in rats (121–123). Some experiments are usually finished with a period of fasting before the sacrifice; but this condition changes the expression of PGC-1*_α_*, AMPK or PPAR, particularly in rodent muscle (124–126).

### Animals

Variations in age, sex, weight, or handling procedures might change both biological and behavioral results.

#### Age

In our analysis, only 38.9% (21/54) of the studies described the age of the animals at the beginning of the training protocol (**Figure 3**). Humans and rodents have adolescence, adulthood, or old age periods of life with their own physiological responses. However, these stages of life are significantly shorter in mice and rats and, therefore, reporting the age of the rodents becomes crucial (27). From birth to adulthood, the rodent’s brain increases in size, myelinization (limbic structures are fully developed at six weeks) and remodels its neural networks (127–129). An age-related mitochondrial decline, hearing loss or changes in liver gene expression with high impact in pharmacological or behavioral responses can be observed in mice (130–132). Also, in stroke models (one of the most common models in forced wheel studies), younger animals may respond differently from older animals (133, 134). Due to the mentioned impact of age on the different physiological responses, describing the age of all animals throughout the experiment should be mandatory.

#### Sex

The 88.9% (48/54) of the studies included in this review described the sex of the rodents (**Figure 3**). The last decades of experimental research have underlined the need of taking into account the differences between male and female rodents (23, 135). Sex differences in hormone levels may affect decision-making mechanisms (136–138). Furthermore, drug effects and therapies, such as physical exercise, show variations between males and females (139–141).

#### Weight

The 88.9% (48/54) of the studies described the weight at some point of the research work, but only 63% (34/54) of them measured it regularly (**Figure 3**). The body weight of the animal may be related to health status and is strongly influenced by numerous nutritional, environmental, husbandry and genetic factors (30, 142–144). Some studies use body weight to deduce the age. However, both age and weight need to be reported together since body weight cannot predict age with precision (145, 146).

#### Handling

Although all the analyzed studies involved some form of manipulation and contact with the rodents, only 9.3% (5/54) described a handling protocol (**Figure 3**). The levels of anxiety and stress of the rodents can be reduced with handling procedures (20). In addition, it can help to manipulate the rodents more calmly, reducing possible bites, and with strong repercussions in behavioral tests (e.g. open field) (147, 148).

### Exercise

To ensure reproducibility of exercise protocols, it is essential to include details of at least the light/dark cycle, habituation protocol and training parameters.

#### Exercise and Light/Dark Cycle

**Figure 4:**
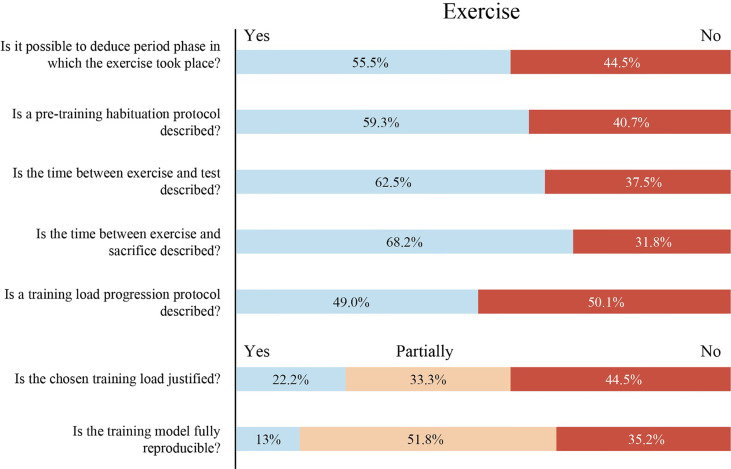
Percentage of studies that reported each item in relation to Exercise. Light blue box: Item reported. Orange box: Item partially reported. Red box: Item not reported.

In this review, in 55.5% (30/54) of the published works it was possible to know whether the exercise took place in the active or passive phase (**Figure 4**). Of these studies, 14 developed the exercise program during the active phase and 13 during the passive phase, without justifying the reasons for the choice. Surprisingly, only 3 studies carried out the training during the inactive or active/inactive period under justified experimental reasons. The beginning of the light phase (passive) is considered the zeitgeber time 0 (ZT0). The use of this nomenclature would allow to avoid confusing comparisons of research works using the time of the day as a reference (149). Because almost all experimental research is developed in nocturnal rodents, it is a priority to point out if the experiments were developed during their active (night) or passive phase (day) (150). Exercise may also re-entrain circadian rhythms, producing a misalignment that can lead to low cognitive performance, deterioration of alertness, weight variations and sleep disruption (33, 151–157). Furthermore, forced activity during the inactive phase may disrupt gene expression patterns and hormonal regulations (e.g. insulin, testosterone or cortisol) (158, 159). Knowing the precise light/dark cycle parameters in which the exercise was developed, major misinterpretations of the research output could be avoided.

#### Habituation Protocol

In this review, 59.3% (32/54) of the analyzed papers described some habituation period before the exercise program (**Figure 4**). Around 10% of rodents refuse to run in forced running paradigms (160). However, that lack of response can be solved by applying a habituation phase prior to an exercise program (18). This period improves the capacity of rodents to maintain a higher volume and intensity of running and establishes a homogeneous starting point for all the animals to perform a training program (18, 161). In addition, the habituation protocol reduces non-specific stress responses in rats that can modify the physiological and behavioral results in forced models (19).

#### Training Parameters

Duration, starting time, speed and frequency of the exercise sessions are key parameters to be reported in order to ensure the reproducibility of exercise protocols. Surprisingly, only 13% (7/54) of the analyzed exercise protocols are completely reproducible (**Figure 4**). Reproducibility was assessed attending to the following factors: (i) duration of each exercise session: specification of series and interspersed rest time (if applies) (min) (ii) speed (m/min), (iii) start time of each exercise session (ZT time), (iv) daily and weekly frequency and (v) total duration of the exercise program (days/weeks). **Supplementary File 3**, Figure 1 from Toval et al. (18) and Table 1 in Toval et al. (19) are examples where all the mentioned training parameters are described. Reporting these parameters is especially relevant since intensity, volume, and density are the three main factors of the training load and changes in all or some of them can produce different adaptations in the organism (31, 162, 163). We encourage authors to use this table (**Supplementary File 3**), implementing the required modifications according to the needs of each study.

#### Load Progression *vs* Non Progression Protocol

Of the papers that developed more than one training session (51/54), only 49% (25/51) applied a load progression protocol (**Figure 4**). The implementation of a load progression (intensity, volume, and density of the exercise) during the training program is one of the main training principles (164). A load progression protocol ensures that new adaptations are occurring in the body throughout the weeks of training (165). Therefore, a failure to implement a load progression protocol might produce misinterpretations of the exercise effects.

#### Training Load Justification

In this review, 44.5% (24/54) of the studies did not justify any load parameter (**Figure 4**). However, 33.3% (18/54) of them reported some parameters while only 22.2% (12/54) reported some justification for all parameters of the exercise load. The training load (exercise intensity, volume and density) to be developed throughout the experimental research needs to be determined by physiological references (e.g. %VO2Max and lactate thresholds) or be based on well-founded work. Intensity, volume or density can strongly modify the research output, and should be reported and taken into consideration carefully (162, 166). In this sense, we observed a lack of justification among the training protocols.

#### Time Between Exercise and Test/Sacrifice

In this review, only 62.5% (15/24) of the studies reported the time between exercise and tests, and 68.2% (30/44) reported the time between exercise and sacrifice (**Figure 4**). Adjustments and adaptations to exercise are produced during an exercise program. Adjustment refers to the short-term changes that occur as a result of increased metabolic demand during the physical exercise (e.g. increased heart rate), while adaptation refers to residual changes in the organism after several exercise sessions (167). Furthermore, these adjustments and adaptations to exercise have an effect on early and late gene expression responses in a time-of-day-dependent way (150, 168). After 1 hour of exercise, the transcriptomic and metabolomic patterns analyzed every 4 hours showed different molecular profiles in a range of 20 hours (168). Thus, establishing and reporting the time between the last session of exercise and any test or analysis (sacrifice) is crucial.

### Study Distribution of the Reported Items and Its Correlations With the Impact Factor and Year of Publication

**Figure 5:**
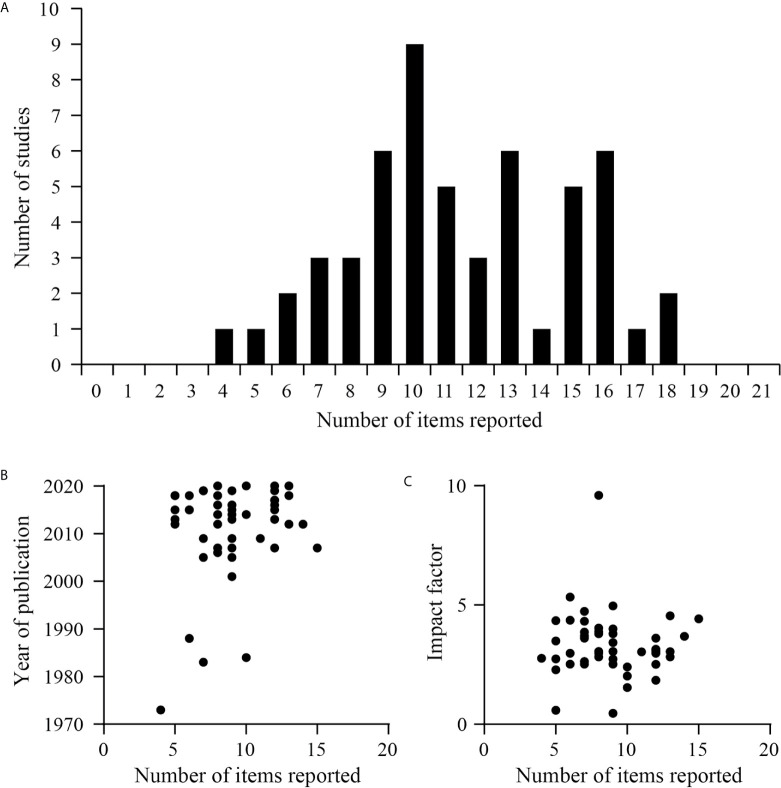
Studies distribution and correlation of the reported items. **(A)** Histogram representing the distribution of studies according to the items reported by them. **(B)** Correlation between the number of items reported by each study and the year of publication of the study in the journal (p=0.28, r=0.15, Pearson’s correlation coefficient r). **(C)** Correlation between the journal’s impact factor (2019) and the number of items reported by each study (p=0.69, r=-0.05, Pearson’s correlation coefficient r).

A histogram was used to determine how the papers were distributed according to the number of the items reported (**Figure 5A**). Items that described weight, training parameters, training load justification and time between exercise and test/sacrifice include an extra point if the items were fully reported, thus being 21 the highest number that could be reached only in entirely detailed works. In our study, a range between 4 and 18 items were reported for the articles analyzed (**Figure 5A**). However, an average of 11.42 ± 3.46 items reported indicates that most of the articles are not detailed enough to be reproducible. Next, we found that the year of publication and the current impact factor are not correlated with the number of items correctly reported in these research works (**Figures 5B, C**; p>0.05), being the correlation defined as small (r=0.15) and trivial (r=-0.05) respectively.

A strong tendency to claim the reporting of critical data to ensure experimental reproducibility began twenty years ago and led to guidelines to overcome these difficulties (1, 3, 4). Our results raise concerns about the lack of transparency in research reports once again (5, 6). Also, the absence of key information, sometimes related with lack of requirement, could not be linked to the year of publication of the research work (1, 2, 95). Furthermore, the imprecise details of the methods did not depend on the impact factor of the journals, the latter being a measure of the quality of the journal questioned by current criticism (169–171).

### FORCED Exercise Wheel Guidelines

After analyzing the degree to which each item mentioned above was reported, we suggested guidelines to facilitate reproducibility, effectiveness and greater transparency of forced-wheel intervention studies (**Table 1**). The resulting checklist consists of 13 items grouped by (i) Ethic committee, (ii) Housing, (iii) Animals, and (iv) Exercise. Additionally, FORCED guidelines may be used to evaluate other works, in which case, each item will be evaluated as follows: (i) Complete, if the item and sub-items (if any) are described; (ii) Partial, when half or more, but not all of the sub-items have been described; (iii) Absent, when less than half of sub-items have been described. Some parameters analyzed in ethic committee, housing or animal sections are included in ARRIVE guidelines. However, these indications may not be enough to ensure the reproducibility of studies using forced exercise in rodents. We hope that our suggested guidelines will serve as a starting point to open processes with an international and consensus-based approach, in the interest of reproducibility in this type of research.

**Table 1:** FORCED guidelines for authors.

Factor	Item	Recommendation	Section/paragraph
Ethical statement	1	Indicate the ethical committee permissions and national or institutional guidelines for the care and use of animals.	
Housing			
Temperature and humidity	2	Indicate environmental conditions of all the rooms containing animals throughout the experiment: a) Temperature b) Relative humidity (%)	
Light/dark cycle	3	Give details of the light/dark cycle indicating: a) The start and end time of the light/dark period. b) The period (light or dark) at which all the experiments/tests are performed.	
Animals per cage	4	Indicate the number of animals per cage, reporting (if applies) any changes in the number of animals throughout the experiment.	
Diet (food/drink)	5	Give details of the diet of the animals during the experiment: a) Macronutrient composition of the diet in percentage. b) Drink characteristics (e.g. water, sucrose (%), ethanol…). c) Availability of food and drink throughout the experiment (e.g. ad libitum or fasting period).	
Animals			
Age	6	Give details of the age (in days) of the animals at the start of the experiment.	
Sex	7	Indicate the sex of the animals. If only one sex is included, explain why.	
Weight	8	Provide details of the body weight at the beginning of the experiment and its evolution during the experiment.	
Handling	9	Provide a detailed protocol of handling (e.g. duration, time and frequency) and the number of experimenters who will have contact with the animals throughout the study.	
Exercise			
Exercise and light/dark cycle	10	Report the start and end time of each training session, indicating whether it corresponds to the active or passive phase of the rodent. Ideally using zeitgeber time (ZT).	
Habituation	11	Provide (if applies) a habituation protocol to exercise (e.g. handling, adaptation to light/dark cycle and training).	
Training parameters	12	Provide a detailed protocol (ideally with a figure) of each habituation and training session, detailing: a) Volume (m), intensity (m/s) and density (rest time between series in minutes) of each session. b) Establishment of a load progression throughout the experiment. c) Indicate if the training load chosen is justified with any internal load parameter (e.g. % of VO2max, lactate thresholds or rely on previous work showing internal load). d) Report criteria for deciding when exercise should be terminated if the animal is not able to continue the programmed training.	
Time between exercise and test/sacrifice	13	a) The time between the end of the last exercise session and the test (if applicable). b) The time from the end of the last exercise session and sacrifice (if applicable).	

## Strengths and Limitations

### Strengths

The systematic review was developed according to PRISMA guidelines. The data extraction manual was developed before field work. The risk of bias of the studies was assessed using SYRCLE’s tool. Two independent reviewers applied the exclusion/inclusion criteria, data extraction and the risk of bias assessment, looking for consensus with a third author in case of disagreements.

### Limitations

The protocol of the review was not registered in databases such as PROSPERO, since it is required to register it prospectively and the field work had already been initiated. A meta-analysis of the included studies was not performed as the aim of the review was to analyze the characteristics that can affect the reproducibility of forced wheel training protocols, instead of the measurement of a treatment effect.

## Conclusion

This systematic review was developed in order to know the reproducibility and reliability of the studies using forced wheel systems in rodents. We concluded that most of the analyzed works do not provide enough data to guarantee the experimental reproducibility and research output comparisons. Our suggested FORCED guidelines are expected to a) be considered to promote a consensus in the field of exercise, b) to be used for *in vivo* experiments with rodents in forced wheel exercise, and c) extended to other modalities such as treadmill exercise. If the variables mentioned by these guidelines are not accurately described, the reported effects of the exercise could be questioned. Our study reaffirms the need for improved reporting in animal research using forced wheel exercise programs. This task can strongly contribute to the experimental reproducibility in this field, and should be carefully considered by authors, editorial boards, and ethics committees.

## Data Availability Statement

The original contributions presented in the study are included in the article/**Supplementary Material**. Further inquiries can be directed to the corresponding author.

## Author Contributions

All authors contributed to the study conception and design. YK, AT, DG, and JF elaborated the extraction manual. DG and MM-M carried out the phases of database searching, screening, and data extraction. Disagreements were revised with AT and AB. DG and AT conducted the risk of bias assessment. Disagreements were revised with AB. DG and JF drafted the initial manuscript. FN-M and BR critically reviewed the manuscript. All authors revised the manuscript and approved the final text as submitted.

## Funding

Granted by the Spanish Ministry of Science, Innovation and Universities (MCIU), State Research Agency (AEI) and European Regional Development Fund (FEDER; PGC2018-098229-B-100 to JF), and by Seneca Foundation (19904/GERM/15).

## Conflict of Interest

The authors declare that the research was conducted in the absence of any commercial or financial relationships that could be construed as a potential conflict of interest.
