# Supplementary material for: A Handful of Details to Ensure the Experimental Reproducibility on the FORCED Running Wheel in Rodents: A Systematic Review
Source: Front Endocrinol (Lausanne). 2021 May 10;12:638261. doi: 10.3389/fendo.2021.638261 (PMC8141847; doi:10.3389/fendo.2021.638261)
Supplement: Supplementary file 2 [file Table_2.docx]

**Supplementary File 2**. Summary of the characteristics of the animals and the exercise intervention of each included study.

| **Authors** | **Animal model** | | | **Exercise protocols** | | | |
| --- | --- | --- | --- | --- | --- | --- | --- |
|  | **Species** | **Strain** | **Healthy/Disease model/Pathology** | **Habituation** | **Nº of training days** | **Frequency** | **Session(s) Load** |
| Arnold et al. (41) | Rat | Fischer | Healthy | Yes | 30 | 5d/wk | Brief bouts of running (average of 2.04 ± 1.95 min) at various speeds (range 4–17 m/min) interspersed with frequent periods of no running (range 0.33–30 min). |
| Biondi et al. (81) | Mouse | C57BL/6J (Janvier) | B6.A/J-Dysf prmd (Bl.A/J) dysferlin-deficient / Dysferlinopathy | No | 20 | 5d/wk | 30’ at 14 m/min |
| Boersma et al. (42) | Rat | Roman Low/high avoidance | RLA (insulin resistant) | Yes | 18 | 7d/wk | 5’ running/5’ rest all day varying speed (max. speed 20m/min) |
| Cao and Picklo (43) | Rat | Sprague-Dawley (SD) | Obese prone / Obesity | Yes | 60 | 5d/wk | 2h at 4m/min and gradually progressed to 8.7 m/min over a 7-week period. Then, rats were maintained at this level of activity for a 5-week period. |
| Caton et al. (44) | Rat | Wistar | Healthy | No | 21 | 7d/wk | 25’ from 10m/min to 14m/min |
|  |  |  |  | No | 21 | 7d/wk | 10’ at 10m/min reaching 12 m/min for 30’ |
| Chen et al. (45) | Rat | Wistar | Stroke | Yes | 15 | 5d/wk | 30’ at 20 m/min (wk1), 30’ at 30 m/min (wk2), 60 min at 30 m/min (wk3) |
| Chhaya et al. (46) | Rat | SD | Spinal Cord Injury (SCI) | Yes | 20 | 5d/wk | 5 m/min with speed increasing at 1’ intervals according to the forelimb capabilities of the rats or until a maximum speed of 14.0 m/min was achieved |
| Detloff et al. (47) | Rat | SD | Spinal Cord Injury | Yes | 4 and 24 | 5d/wk | 5 m/min with speed increasing at 1’ intervals according to the forelimb capabilities of the rats or until a maximum speed of 14.0 m/min was achieved |
| Eccles et al. (48) | Rat | SD | Healthy | No | 6 | 6d/wk | 30 min at 70 revolutions |
| Forristall et al. (49) | Rat | SD | Healthy | Yes | 4 | 4d/wk | 30' at 147.0 (15.63) revolutions per minute (RPM) |
|  |  |  |  | Yes | 5 | 5d/wk | From 135 to 199.5 RPM in 5 days |
| Garcia et al. (82) | Mouse | C57BL/6J (Jackson) | Healthy | Yes | 4 | 4d/wk | 60’ at start speed of 2.5 m/min, which was increased by 0.3 m/min, every 10’ / Speed began at 2.5 m/min and increased 0.3 m/min every 10 min until the mouse reached a Tc of 41°C. |
| Greenwood et al. (50) | Rat | Fischer 344 | Lesion in mPFC | Yes | 30 | 5d/wk | Brief bouts of running (average of 2.04 ± 1.95 min) at various speeds (range 4–17 m/min) interspersed with frequent periods of no running (range 0.33–30 min). |
| Griesbach et al. (51) | Rat | SD | FPI in Left parietal cortex | No | 10 | 5d/wk | 20’ at similar velocity that voluntary wheel running group / 20’ at similar velocity that voluntary wheel running group |
| Hagar et al. (83) | Mouse | BALB/c | Athymic | Yes | 40 | 5d/wk | When a mouse would show first signs of exhaustion by freezing or clinging to the rungs, the velocity would be lowered until the mouse would begin running again. In the 8th and final week, 26’ with 1 min at 6 m/min, 1 min at 8 m/min, 22 min at 10 m/min, and 2 min 12 m/min. |
| Ilback et al. (52) | Rat | SD | Salmonella infection | No | 1 | 1d/wk | 120’ at 10 m/min |
| Ji et al. (53) | Rat | SD | Healthy | No | 15 | 5d/wk | 30’ at 6, 8 and 10 rounds/min / 30’ at 6, 8 and 10 rounds/min |
|  |  |  | Irradiated (brdu) | No | 15 | 5d/wk | 30’ at 6, 8 and 10 rounds/min / 30’ at 6, 8 and 10 rounds/min |
| Kang et al. (84) | Mouse | C57BL/6J (Jackson) | Healthy | Yes | 80 | 5d/wk | 1h at 7 m/min |
| Kant et al. (54) | Rat | SD | Stress | No | 10 | 7d/wk | 15’ at 8 RPM (diameter=38 cm) |
| Kant et al. (55) | Rat | SD | Stress | No | 1 | 1d/wk | 5, 15 or 60’ (prior to sacrifice) at 8 RPM (diameter=38 cm) |
| Kennard et al. (85) | Mouse | C57BL/6J (Jackson) | Healthy | Yes | 25 and 50 | 5d/wk | Low impact: 45’ speeds progressively increasing to a maximum of 10 m/min (two sessions per day). High impact: 45’ with speeds progressively increasing to a maximum of 21 m/min (two sessions per day). |
| Kim et al. (86) | Mouse | C57BL/6J (Daehan BioLink) | Hcrt/Orx/MCH KO | Yes | 21 | 7d/wk | 1 h at 9 m/min |
| Kim et al. (87) | Mouse | C57BL/6J (Daehan BioLink) | Stress | Yes | 21 | 7d/wk | 1 h at 9 m/min |
|  |  |  | Stress | Yes | 7 | 7d/wk | 1 h at 9 m/min |
| King et al (88) | Mouse | C57BL/6J (Jackson) | Stroke | Yes | 4 | 4d/wk | 60’ at start speed of 2.5 m/min, which was increased by 0.3 m/min, every 10’. In the 4th and final session, exercise time and incremental speed were elevated until the animals exhibited fatigue. |
| Laitano et al. (89) | Mouse | C57BL/6J (Jackson) | Stroke | Yes | 4 | 4d/wk | 60’ at start speed of 2.5 m/min, which was increased by 0.3 m/min, every 10’. In the 4th and final session, exercise time and incremental speed were elevated until the animals exhibited fatigue. |
| Lloyd et al. (56) | Rat | Fischer 344 | Healthy | Yes | 30 | 5d/wk | Brief bouts of running (average of 2.04 ± 1.95 min) at various speeds (range 4–17 m/min) interspersed with frequent periods of no running (range 0.33–30 min). |
| Mancardi et al. (57) | Rat | Wistar | Healthy | No | 20 | 5d/wk | Wheel radius (25cm) from 5 to 12 laps per min and running duration (from 10 to 30 min) to accommodate increased fitness (mean velocity 15-30 cm/s circa). The highest level of activity [12 laps/min (i.e. 30 cm/s) for 30 min] included a 5-min warm-up period at 5 laps/min,  without a final period of warm down. Increase in velocity  and/or duration were performed when the rats were able  to maintain the exercise intensity for 2-3 consecutive sessions. |
| Martinez-Salazar (58) | Rat | Wistar | Hypothyroidism | No | 20 | 5d/wk | 30’ at 5m/min |
| Masaki and Nakajima (59) | Rat | Wistar | Healthy | No | 3 | 3d/wk | 60’ at 8m/min |
| Nakajima (60) | Rat | Wistar | Healthy | No | 4 | 4d/wk | Two cycles of a 4-day baseline and a 4-day running treatment, and the test sequence was ended with a 2-day baseline. The speed of rotation was increased across the 3 running phases (98, 185, and 365 m/h) for the first squad of rats (ascending rats), while it was decreased (365, 185, and 98 m/h) for the second squad (the descending rats). |
|  |  |  | Healthy | No | 4 | 4d/wk | 1h at 80 m/h |
| O’Dell et al. (61) | Rat | SD | Nigrostriatal dopamine injury 6.OHDA | Yes | 12+10 | - | Day 1, running at 3.5 m/min for 5 min, twice a day, with gradual increases in the speed and duration of the sessions occurring each day until a criterion of 10 m/min for each of two 30 min sessions was reached. On day 8, the animals were housed with voluntary running wheels, where they were allowed ad libitum exercise in addition to the twice-daily forced exercise periods. After surgery, 6 m/min for 10 min twice a day. The speed and duration were gradually increased until they re-attained 30’ at 10m/min (two sessions) |
| Patel and White (62) | Rat | Lewis | Experimental autoimmune encephalomyelitis | Yes | 10 | 7d/wk | Exercise bouts began with 30 min of running at 15 m/min, then increased to 30 m/min for the remaining time. 60’ on days 1 and 2 and 90’ on days 3 to 10. |
| Peng et al. (63) | Rat | Wistar | Periprosthetic inflammation and osteolysis | No | 100 | 5d/wk | 500m in 90 to 120’ |
| Pianta et al. (64) | Rat | SD | Stroke | No | 1 | 1d/wk | 30 min (30m) exercise group ran at a speed of 2 m/min for the first 5 min, 5 m/min for the next 5 min, and 8 m/min for the remaining 20 min. The 60 min (60m) exercise group ran at a speed of 2 m/min for the first 5 min, 5 m/min for the next 5 min, and 8 m/min for the next 20 min, 2 m/min for the next 5 min, 5 m/min for the next 5 min, and 8 m/min for the remaining 20 min |
| Picklo and Thyfault (65) | Rat | SD | Obese prone/Obesity | No | 60 | 5d/wk | 4m/min for a total of 200 m/day and progressed to 8.7 m/min for a total 1200 m/day, over a 7-week period |
| Ploughman et al. (66) | Rat | SD | Focal ischemia | Yes | 1 | 1d/wk | 30’ or 60’ at 11m/min or 30’ at 14 m/min |
| Ploughman et al. (67) | Rat | SD | Focal ischemia | Yes | 1 | 1d/wk | 60’ at 11 m/min |
|  |  |  |  | Yes | 1 | 1d/wk | 60’ at 11 m/min (two sessions) |
| Ranjbar et al. (90) | Mouse | BALB/c | Tumor | Yes | 39 | 7d/wk | 25’ from 5 to 9 m/min |
| Rezaei et al. (68) | Rat | Wistar | Healthy | Yes | - | - | Different test to determine lactate thresholds:  LT (Lactate threshold): The test began at the speed of 2.5 m/min, with an increase of 2.5 m/min at the end of every 3-min stage. The speed at which rats changed their movement pattern (five times in 3 min), repeatedly grabbing the wheel and refraining from running, was regarded as the exhaustion speed.  MLSS (Maximal Lactate Steady State): The MLSS was considered as the highest intensity of the constant power test with less than 1 mM increase in blood lactate concentration between 10th and 25th min. The MLSS concentration was calculated as the mean blood lactate concentration measured at 10, 15, 20, and 25 min of test. |
| Saito et al. (69) | Rat | Wistar | Healthy | No | 1 | 1d/wk | 10’ at 6 rpm (7.7 m). |
| Sandrow-Feinberg et al. (70) | Rat | SD | SCI | Yes | 20 | 5d/wk | Start 5  days after injury with a beginning speed of 5m/min, with  wheel speed increased daily according to the fore limb capabilities  of individual animals to a maximum speed of  14.0m/min |
|  |  |  |  |  |  |  |  |
| Sasaki et al. (91) | Mouse | - | PER2::LUC KO | No | 3 or 10 | 3 or 7d/wk | 90’ at 8m/min |
| Smith et al. (71) | Rat | SD | Cingulotomy | No | 1 | 1d/wk | 10’ (speed not reported) |
| Smith et al. (72) | Rat | Long-Evans | Healthy (cocaine adicction) | Yes | 3 | 3d/wk | 30’ at 12m/min |
| Spurgeon et al. (73) | Rat | Wistar | Healthy (old) | No | 90-110 | 5d/wk | 30’ at 11.6m/min |
| Stevenson et al. (74) | Rat | Long-Evans | Healthy | Yes | 1 | 1d/wk | 500 or 1000m at 9m/min |
| Stoyell-Conti et al. (92) | Mouse | C57BL/6J (Jackson) | Healthy | Yes | 40 | 5d/wk | 1h from 2m/min to 5m/min |
| Tang et al. (93) | Mouse | C57BL/6J (Jackson) | Colitis | No | 10 | 7d/wk | 24h at 1 round per minute (diameter = 13.5 in.) |
| Toval et al. (18) | Rat | SD | Healthy | Yes | 8 | 7d/wk | Day 1: 2’ at 5m/min. Day 2: 5’ at 7.2m/min. Day 3: 10’/5’rest at 7.2m/min. Day 4: 2x10’/5’ rest at 8.1m/min. Day 5: 3x10’/5’ at 8.1m/min. Day 6: 2x10’/5’rest at 9m/min (two sessions). Day 7: 3x10’/5’rest at 9m/min |
| Tsai and Tsai (75) | Rat | F344 | Healthy | Yes | 91 | 7d/wk | 84 revolutions/min in bouts during 12h |
| Wang et al. (76) | Rat | SD | Parkinsonism (6OHDA) | Yes | 20 | 5d/wk | 20’ at varying speed, depending on whether or not the rodent adapts to the new speed |
| Wang et al. (77) | Rat | SD | Parkinsonism (6OHDA) | Yes | 20 | 5d/wk | 20’ at varying speed, depending on whether or not the rodent adapts to the new speed |
| Whishaw and Vanderwolf (78) | Rat | Hooded | Healthy | Yes | 1/ - | - | Ten rats were gradually adapted to running in a 183-cm circumference motor-driven wheel at a speed of 33 ft/min. They were then given a single continuous 8-hr session of running. Two of the rats continued running 6-9 hr/day until they had run a total of 100 hr. Five remaining rats were trained to run at speeds of 33, 60, 90 and 144 ft/min. |
| Zhang et al. (79) | Rat | SD | Stroke-prone renovascular hypertensive model | No | 40 | 5d/wk | 30’ in total: 10’ at 5m/min, 10’ at 7m/min and 10’ at 9/min. |
| Zhang et al. (80) | Rat | SD | MCAO (Middle Cerebral Artery Occlusion) | No | 14 | 7d/wk | 20’ at 2m/min twice a day, then gradually  increased to 3.25m/min on the third day and 6.59m/min on the seventh day post-MCAO |
|  |  |  |  | No | 15 | 7d/wk | High intensity: 20’ at 3.25m/min, at 48 hr;  5.3m/min, at the 3rd day; 8.45m/min, at the 7th day after MCAO.  Moderate intensity: 20’ at 2m/min twice a day, then gradually  increased to 3.25m/min on the third day and 6.59m/min on the seventh day post-MCAO.  Low intensity: 20’ at 0.66m/min, at 48 hr; 2m/min, at the 3rd day; 4.62m/min at the 7^th^ day after MCAO. |
