# Supplementary material for: A Handful of Details to Ensure the Experimental Reproducibility on the FORCED Running Wheel in Rodents: A Systematic Review
Source: Front Endocrinol (Lausanne). 2021 May 10;12:638261. doi: 10.3389/fendo.2021.638261 (PMC8141847; doi:10.3389/fendo.2021.638261)
Supplement: Supplementary file 3 [file Table_3.doc]

**Supplementary File 3.** Example template on how to report all exercise parameters needed for reproducibility

|  | **HANDLING^1^** | | | | | |  |  |  |  | | | | | | | | | | | | | | | | | | | | | | | | | | | |
| --- | --- | --- | --- | --- | --- | --- | --- | --- | --- | --- | --- | --- | --- | --- | --- | --- | --- | --- | --- | --- | --- | --- | --- | --- | --- | --- | --- | --- | --- | --- | --- | --- | --- | --- | --- | --- | --- |
| Session no.^2^ | **-5** | **-4** | **-3** | **-2** | **-1** | **0** |  |  |  |  | | | | | | | | | | | | | | | | | | | | | | | | | | | |
| Postnatal Day^3^ | P20 | P21 | P22 | P23 | P24 | P25 |  |  |  |  | | | | | | | | | | | | | | | | | | | | | | | | | | | |
|  | Random. | Adaptation to experimental conditions (room conditions, handling, etc). | | | | |  |  |  |  | | | | | | | | | | | | | | | | | | | | | | | | | | | |
|  |  |  |  |  |  |  |  |  |  |  |  |  |  |  |  |  |  |  |  |  |  |  |  |  |  |  |  |  |  |  |  |  |  |  |  |  |  |
|  | **HABITUATION** | | | | | | | |  | | | | | | | | | | | | | | | | | | | | | | | | | | | |
| Session no. | **1** | **2** | **3** | **4** | **5** | **6** | **7** | **9** |  | | | | | | | | | | | | | | | | | | | | | | | | | | | |
| Postnatal Day | P26 | P27 | P28 | P29 | P30 | P31 | P32 | P33 |  | | | | | | | | | | | | | | | | | | | | | | | | | | | |
| Time | **ZT14^4^** | | | | | | | |  | | | | | | | | | | | | | | | | | | | | | | | | | | | |
| Duration (min)^5^ | 5 | 5 + 2 | 5 | 10 | 2x10 (5'rest) | 3x10 (5'rest) | 2x10 (5'rest) | 3x10 (5'rest) |  | | | | | | | | | | | | | | | | | | | | | | | | | | | |
| Speed (m/min)^5^ | 0 | 0 + 5 | 7 | 7 | 8 | 8 | 9 | 9 |  | | | | | | | | | | | | | | | | | | | | | | | | | | | |
| Time |  |  |  |  |  |  | **ZT20** | |  | | | | | | | | | | | | | | | | | | | | | | | | | | | |
| Session no. |  |  |  |  |  |  | **8** | **10** |  | | | | | | | | | | | | | | | | | | | | | | | | | | | |
| Duration (min) |  |  |  |  |  |  | 2x10 (5'rest) | 3x10 (5'rest) |  | | | | | | | | | | | | | | | | | | | | | | | | | | | |
| Speed (m/min) |  |  |  |  |  |  | 9 | 9 |  | | | | | | | | | | | | | | | | | | | | | | | | | | | |

|  |  |  |  |  |  |  |  |  |  |  |  |  |  |  |  |  |  |  |  |  |  |  |  |  |  |  |  |  |  |  |  |  |  |  |  |  |  |
| --- | --- | --- | --- | --- | --- | --- | --- | --- | --- | --- | --- | --- | --- | --- | --- | --- | --- | --- | --- | --- | --- | --- | --- | --- | --- | --- | --- | --- | --- | --- | --- | --- | --- | --- | --- | --- | --- |
|  | **TRAINING** | | | | | | | | **TEST** |  | | | | | | | | | | | | | | | | | | | | | | | | | | | |
| Session no. | **10** | **11** | **12** | **13** | **14** | **15** | **16** | **18** |  |  | | | | | | | | | | | | | | | | | | | | | | | | | | | |
| Postnatal Day | P34 | P35 | P36 | P37 | P38 | P39 | P40 | P41 | P42 |  | | | | | | | | | | | | | | | | | | | | | | | | | | | |
| Time | **ZT14** | | | | | | | |  |  | | | | | | | | | | | | | | | | | | | | | | | | | | | |
| Duration (min) | 4x10’ (3’ rest) | 6x10’ (3’ rest) | 4x10’ (3’ rest) | 6x10’ (3’ rest) | 4x10’ (3’ rest) | 6x10’ (3’ rest) | 4x10’ (3’ rest) | 5x10’ (3’ rest) | **OPEN FIELD^6^** |  | | | | | | | | | | | | | | | | | | | | | | | | | | | |
| Speed (m/min) | 9 | 10 | 11 | 11 | 15 | 15 | 17 | 20 |  | | | | | | | | | | | | | | | | | | | | | | | | | | | |
| Time |  |  |  |  |  |  | **ZT20** | | |  | | | | | | | | | | | | | | | | | | | | | | | | | | | |
| Session no. |  |  |  |  |  |  | **17** | **19** |  |  | | | | | | | | | | | | | | | | | | | | | | | | | | | |
| Duration (min) |  |  |  |  |  |  | 4x10’ (3’ rest) | 5x10’ (3’ rest) | **SACRIFICE^6^** |  | | | | | | | | | | | | | | | | | | | | | | | | | | | |
| Speed (m/min) |  |  |  |  |  |  | 17 | 20 |  | | | | | | | | | | | | | | | | | | | | | | | | | | | |

^1^Indicates the training phase (e.g. handling, habituation or training).

^2^Indicating the number of the sessions is useful to know hoy many sessions are in total in each phase and to organize the characteristics of each session. Session: Time spent inside the wheel.

^3^Reporting age helps to know precisely what day they start and end each phase of training.

^4^Reporting the starting time (in zeitgebers) of each session.

^5^Indicate the training load of each day (volume, intensity and density) in minutes (volume and rest) and m/min (speed), trying to avoid other forms of measuring (e.g. revolutions per minute) that make difficult to read and reproduce the training load.

^6^Indicating information about the different tests/sacrifice (if any) in the same training schedule could facilitate researchers to know the precise information to reproduce and interpret the analysis and test results.
