# Supplementary material for: A Handful of Details to Ensure the Experimental Reproducibility on the FORCED Running Wheel in Rodents: A Systematic Review
Source: Front Endocrinol (Lausanne). 2021 May 10;12:638261. doi: 10.3389/fendo.2021.638261 (PMC8141847; doi:10.3389/fendo.2021.638261)
Supplement: Supplementary file 4 [file Table_4.docx]

**Supplementary File 4.** Examples of two electronic search strategies.

**Pubmed search. No limitations.**

("forced running wheel*"[Title/Abstract] OR "forced wheel*"[Title/Abstract] OR "motorized wheel*"[Title/Abstract])

AND

(rodent*[Title/Abstract] OR rat[Title/Abstract] OR rats[Title/Abstract] OR mice[Title/Abstract] OR mouse[Title/Abstract] OR mus[Title/Abstract] OR murine[Title/Abstract])

**Web of science search. No limitations.**

Topic: (("forced running wheel*" OR "forced wheel*" OR "motorized wheel*")

AND

(rodent* OR rat OR rats OR mice OR mouse OR mus OR murine))
