# Supplementary material for: A Handful of Details to Ensure the Experimental Reproducibility on the FORCED Running Wheel in Rodents: A Systematic Review
Source: Front Endocrinol (Lausanne). 2021 May 10;12:638261. doi: 10.3389/fendo.2021.638261 (PMC8141847; doi:10.3389/fendo.2021.638261)
Supplement: Supplementary file 5 [file Table_5.docx]

**Supplementary File 5.** Variables extraction protocol.

**Variables explanation**

**Procedures**

The data extraction will be done in Microsoft Excel, using a data matrix with columns and rows. All dichotomous variables whose only answer is "yes/no" will be coded as follows: 0=no; 1=yes. They will be accompanied by # in the description. If a variable is not specified in the study (but should be), it will be coded as "x". All those variables that do not apply due to the methodology or objectives of the original studies will be filled in with "n".

**Variables to be analyzed in the studies:**

**Organizational variables**

| **Column**  **(Animals)** | **Name** | **Description** |
| --- | --- | --- |
| **A** | **#Paper** | The number assigned to the study. Each document has a unique number. |
| **B** | **Letter** | The letter corresponding to the experiments carried out in the studies. Thus, a multi-experimental study that has 4 groups (2 control and 2 experimental), will have 2 experiments, and therefore will be 1a and 1b. The letters will be assigned in the same order as the experiments are named within the document, but the document will always have the same number (1, in this example). |

**Extrinsic variables**

| **Column** | **Name** | **Description** |
| --- | --- | --- |
| **C** | **LabCountry** | Country where the laboratory is localized. |
| **D** | **Year** | Year in which the study was published. |
| **E** | **Journal** | Journal in which the study is published. |

**Methodological variables**

| **Column** | **Name** | **Description** |
| --- | --- | --- |
| **F** | **ExpGroups** | Number of experimental groups. |
| **G** | **ConGroups** | Number of control groups. |
| **H** | **ExpRepli** | Whether the study has experimental replications or not. # |
| **I** | **NumExpRepli** | Number of experimental replications. |
| **J** | **Random** | Whether the sample is randomized or not. # |
| **K** | **EthicComit** | If the study has been approved by an ethics committee. # |
| **L** | **Temperature** | Whether the study specifies the ambient temperature during the experiment or not # |
| **M** | **Humidity** | Whether the study specifies ambient humidity during the experiment or not. # |
| **N** | **InvCycle** | Whether the cycle is reversed or not. # |
| **O** | **InvCycleHour** | It describes the start time of the light-dark cycle or not. # |

**Animal variables**

| **Column** | **Name** | **Description** |
| --- | --- | --- |
| **Animals** | | |
| **P** | **Order** | Order to which the animal sample belongs. |
| **Q** | **Species** | Species to which the animal sample belongs. |
| **R** | **Strain** | The strain to which the animal sample belongs |
| **S** | **DiseaseModel** | Model used to simulate a disease |
| **T** | **ModelPatho** | Pathology developed of the animal. |
| **U** | **AgePA(d)** | Age in days of the animal sample when they start physical exercise. |
| **V** | **Weight** | Whether or not weight was evaluated. # |
| **W** | **WeightFreq** | Weighing frequency.  0=Once during the experiment  1=At the beginning and at the end  2=Periodically |
| **X** | **nTotal** | Total number of animals in the sample. |
| **Y** | **%MaleTotal** | Percentage of males in the sample. |
| **Housing** | | |
| **Z** | **NumAniCage** | Specify the number of animals per cage or not. # |
| **AA** | **Food/drink** | Type of diet:  0=Not reported  1=Normal ad libitum diet  2=Any other type of diet combination |
| **AB** | **Handling** | Whether or not there has been physical contact with the animal sample or a protocol has been followed for the animals to enter and exit on their own:  0=No contact, no protocol specified.  1=No contact, with specified protocol.  2=With contact, without specified protocol.  3=With contact, with specified protocol. |

**Intervention variables**

| **Column** | **Name** | **Description** |
| --- | --- | --- |
| **Exercise** | | |
| **AC** | **CycleExer** | Whether the animal sample runs during its day or night cycle:  0=Night  1=Day |
| **AD** | **ExerHabitu** | Whether there is an exercise habituation protocol or not. # |
| **AE** | **ExerProtoDur(d)** | Training duration in days. |
| **AF** | **ProgDur(wk)** | Training duration in weeks. |
| **AG** | **DailyFreq** | Daily exercise frequency (number of sessions per day). |
| **AH** | **WeeklyFreq** | Weekly frequency of exercise (days per week). |
| **AI** | **SchedSess** | Time at which the animals start the exercise (24h). |
| **AJ** | **SessLoad1** | Training load characteristics (volume, intensity and density) of first session of the day. |
| **AK** | **SessLoad2** | Training load characteristics (volume, intensity and density) of second session (if any) of the day. |
| **AL** | **SessLoad3** | Training load characteristics (volume, intensity and density) of third session (if any) of the day. |
| **AM** | **TimeBtwEx&Tests** | Is the time from the end of the exercise to the performance of the behavioral tests specified? # |
| **AN** | **TimeBtwTest&Analy** | Is the time in hours from the completion of the tests to the sacrifice of the animal specified? # |
| **AO** | **RestBtwSess** | Rest between exercise sessions. |
| **AP** | **ProgressLoad** | Whether a load progression protocol is described or not. # |
| **AQ** | **TrainingLoad** | They justify the criteria by which they choose the load (intensity, volume and density).  0=Do not justify anything  1=Some parameters are justified, but not all  2=All parameters are justified (intensity, volume and density) |
| **AR** | **ReproducibleProtocol** | Is the exercise protocol completely reproducible in detail?  To be fully reproducible, one must be able to deduce: Volume, intensity, density and complete quantification of the activity of each subject. |
